# Erratum: Jesus, T.S., et al. Global Need for Physical Rehabilitation: Systematic Analysis from the Global Burden of Disease Study 2017. *Int. J. Environ. Res. Public Health* 2019, *16*, 980

**DOI:** 10.3390/ijerph17239065

**Published:** 2020-12-04

**Authors:** Tiago S. Jesus, Michel D. Landry, Helen Hoenig

**Affiliations:** 1 Global Health and Tropical Medicine & WHO Collaborating Center on Health Workforce Policy and Planning, Institute of Hygiene and Tropical Medicine-NOVA University of Lisbon, Rua da Junqueira 100, 1349-008 Lisbon, Portugal; 2 Physical Therapy Division, Department of Orthopeadic Surgery, School of Medicine, Duke University, Durham, NC 27705, USA; mike.landry@duke.edu; 3 Duke Global Health Institute (DGHI), Duke University, Durham, NC 27710, USA; 4 Physical Medicine and Rehabilitation Service, Durham Veterans Administration Medical Center, Durham, NC 27705, USA; helen.hoenig@va.gov; 5 Division of Geriatrics, Department of Medicine, Duke University Medical Center, Durham, NC 27710, USA

The authors wish to add the following correction to their paper published in International Journal of Environmental Research and Public Health [1].

In the heading of Table 3 (page 6) and of Table 4 (page 7), the authors inform that YLD Counts (i.e., Years Lived with Disability) are reported in “*Billions*”; however, that should be “*Millions*”. The numbers inscribed in the YLD Counts are otherwise accurate (i.e., if converted to millions, not billions). 

Additionally, that reporting error in the tables’ heading was reflected in the text of the manuscript and its abstract, as detailed below:

- In the Abstract (page 1) and Results (page 5): reported as “*5.1 billion YLDs per year*”, should be “*5.1 million YLDs per year*”.
- In the Results (page 7): reported as “*225 billion YLDs in 2017*”, should be “*225 million YLDs in 2017*”.
- In the Discussion (page 8): reported as “*5.1 billion additional YLDs per year*”, it should be “*5.1 million additional YLDs per year*”.

It is noteworthy that although the magnitude of the results is different than initially reported, for the given metric (YLD Counts), that does not alter the pattern of results for the evolution over time for any of the metrics. For example, the reported percent growth in YLD Counts from 1990 to 2017 is the same (66.2% for World, Table 3), because the underlying values for 1990 and 2017 are merely different in conversion units. The discussion of the results and related implications, focused on the evolutionary trend in global physical rehabilitation needs, remains applicable. 

Finally, an unrelated, typographical error also was identified, and needs correction:

In the abstract, when it is mentioned that “YLD Rates per 100,000 people and the percentage of YLDs likely benefiting from physical rehabilitation also grew significantly over time, across locations (all p > 0.05)”, the “>” symbol should be “<”.

The authors would like to apologize for any confusion or inconvenience.
