# Supplementary material for: Global Need for Physical Rehabilitation: Systematic Analysis from the Global Burden of Disease Study 2017
Source: Int J Environ Res Public Health. 2019 Mar 19;16(6):980. doi: 10.3390/ijerph16060980 (PMC6466363; doi:10.3390/ijerph16060980)
Supplement: Supplementary file 1 [file ijerph-16-00980-s001.pdf]

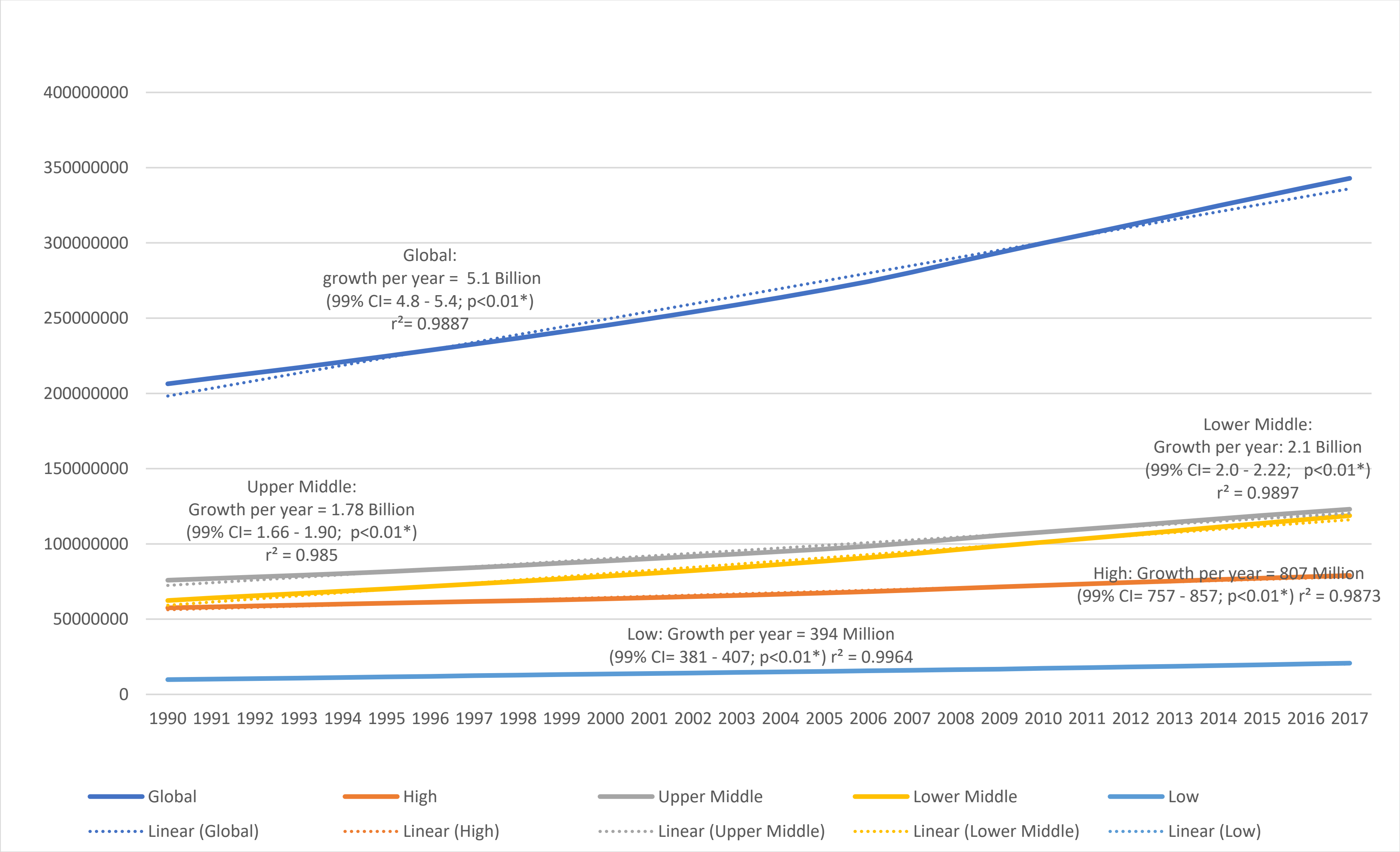

Figure S1. YLD Counts likely benefiting from phyisical rehabilitation along with the "best fit" regression models.

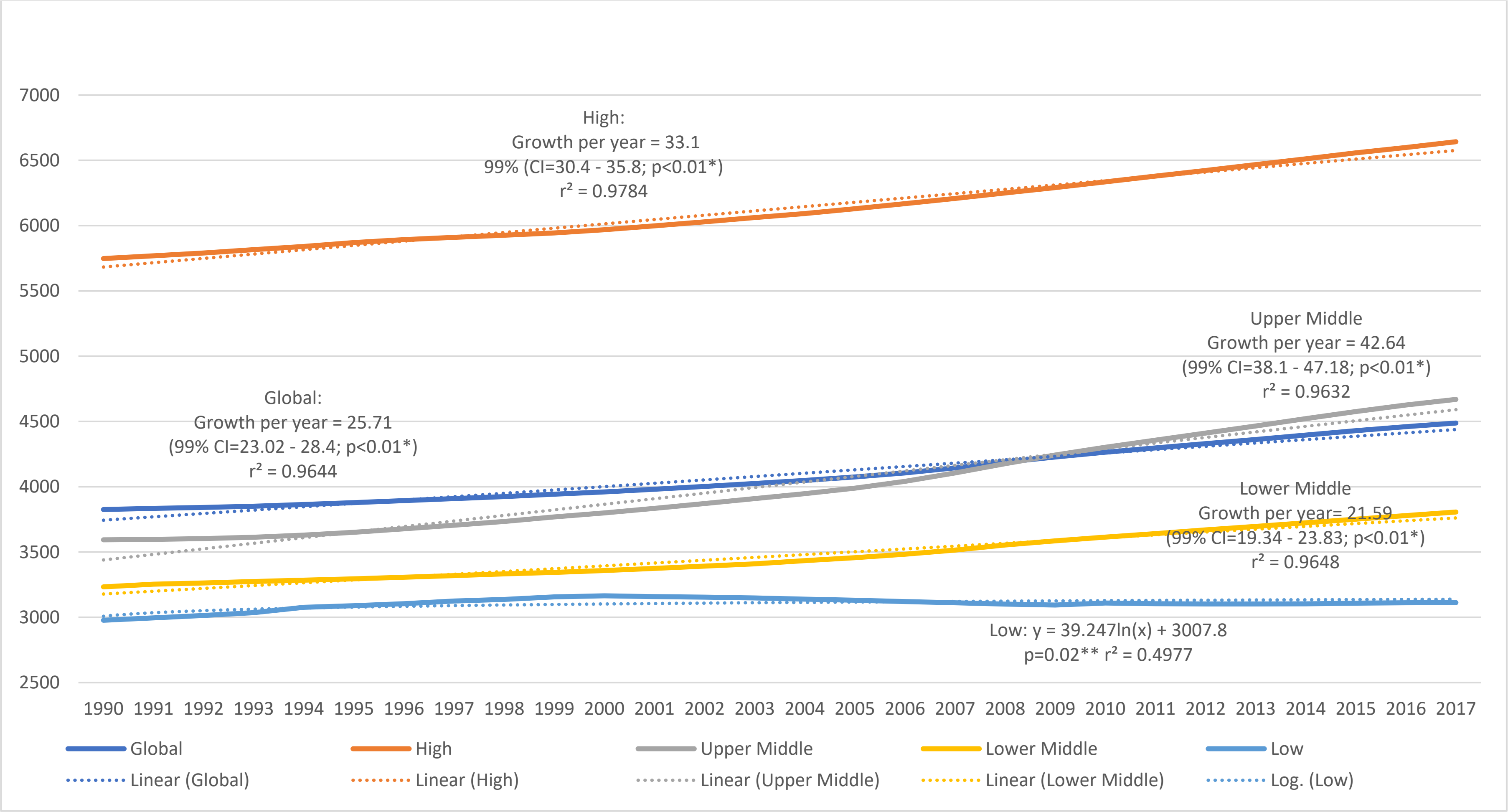

Figure S2. YLD Rates (per 100,000 People) likley benefiting from physical rehabilitation along with the "best fit" regression models.

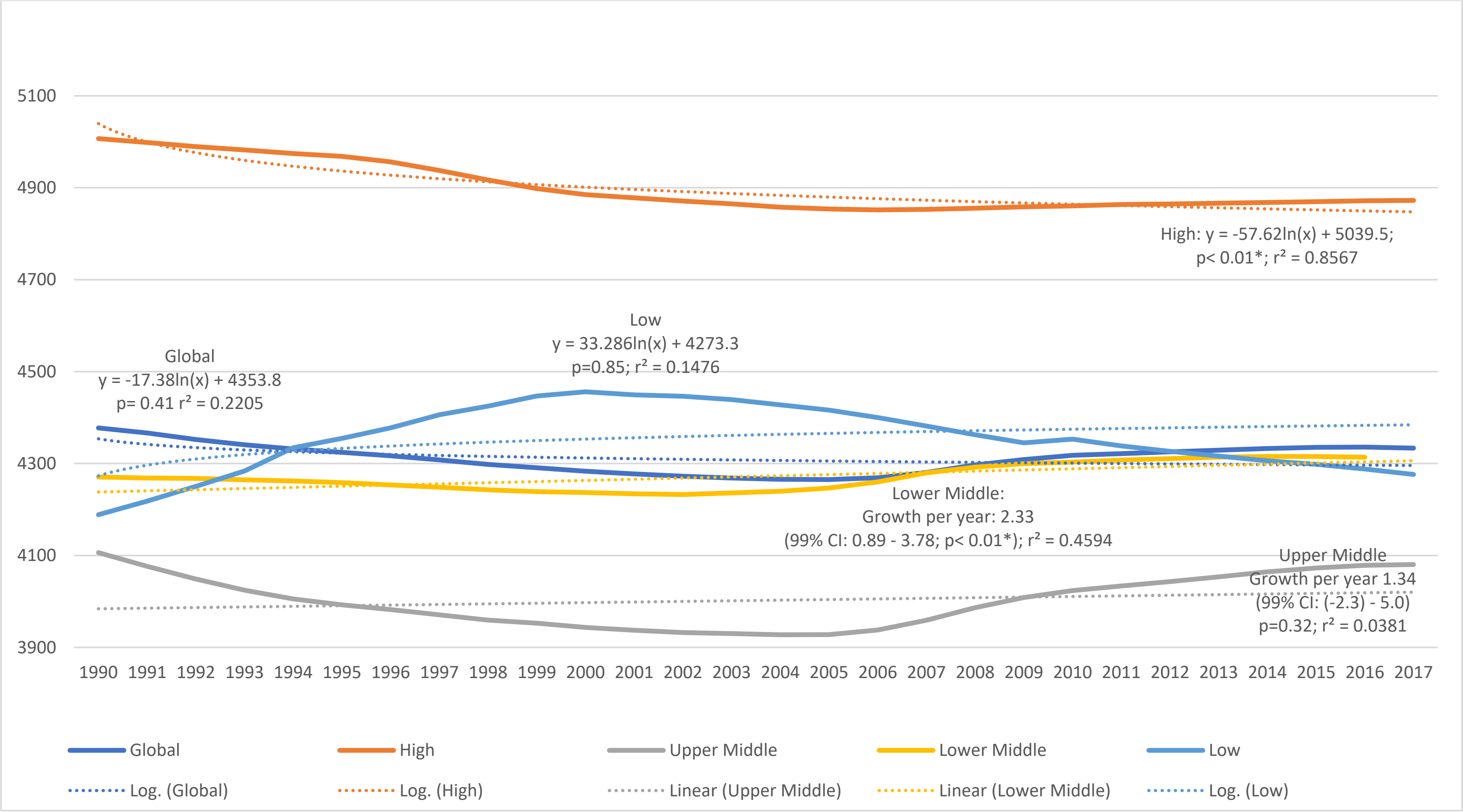

Figure S3: Age-standardized YLD Rates likely benefiting from physical rehabilitation along with the "best fit" regression models.
